# Low-Oxygen Responses of Cut Carnation Flowers Associated with Modified Atmosphere Packaging

**DOI:** 10.3390/plants12142738

**Published:** 2023-07-23

**Authors:** Misaki Nakayama, Nao Harada, Ai Murai, Sayaka Ueyama, Taro Harada

**Affiliations:** 1 School of Education, Okayama University, 3-1-1 Tsushima-naka, Kita-ku, Okayama 700-8530, Japan; 2 Faculty of Education, Okayama University, 3-1-1 Tsushima-naka, Kita-ku, Okayama 700-8530, Japan

**Keywords:** carnation, modified atmosphere packaging, adenylate energy charge, hypoxia-responsive genes, AP2/ERF superfamily

## Abstract

Gaseous factors affect post-harvest physiological processes in horticultural crops, including ornamental flowers. However, the molecular responses of cut flowers to the low-oxygen conditions associated with modified atmosphere packaging (MAP) have not yet been elucidated. Here, we show that storage of cut carnation flowers in a sealed polypropylene bag decreased the oxygen concentration in the bag to 3–5% and slowed flower opening. The vase life of carnation flowers after storage for seven days under MAP conditions was comparable to that without storage and was improved by the application of a commercial-quality preservative. The adenylate energy charge (AEC) was maintained at high levels in petals from florets stored under MAP conditions. This was accompanied by the upregulation of four hypoxia-related genes, among which the HYPOXIA-RESPONSIVE ETHYLENE RESPONSE FACTOR and PHYTOGLOBIN genes (*DcERF19* and *DcPGB1*) were newly identified. These results suggest that hypoxia-responsive genes contribute to the maintenance of the energy status in carnation flowers stored under MAP conditions, making this gas-controlling technique potentially effective for maintaining cut flower quality without cooling.

## 1. Introduction

Gaseous factors, such as oxygen, carbon dioxide, and ethylene, affect various physiological processes and, thus, the postharvest quality of horticultural crops [1,2]. An atmosphere with low oxygen and high carbon dioxide levels suppresses the respiration of plant organs and delays maturation or senescence, leading to the maintenance of harvest quality [3]. In the case of ornamental flowers, some studies have emphasized the deteriorative effects of a controlled atmosphere (CA) [4,5], while others have demonstrated the favorable effects of modified atmosphere packaging (MAP) [6,7,8,9]. Hydration and temperature are the other main determinants of cut flower quality during storage. While wet management keeps active flower opening, dry management for several periods may suppress transpiration and flower opening [6,10]. To increase the effects of storage, cool temperatures are preferred over near-ambient temperatures, but the latter can be employed to reduce costs [6]. These handling methods could affect the vase life of cut flowers, the period between the placement of stems in the vase solution, and the loss of ornamental value. This is key to customer perceived value and, thus, is directly related to the satisfaction gained [11].

Some reports have described the physiological responses of cut flowers to low oxygen levels, especially in carnations, which are typical climacteric flowers that show ethylene-dependent flower senescence. The energy status evaluated by the adenylate energy charge (AEC) [12] is maintained in carnation petals under 5% oxygen but not under anoxia [13]. Under hypoxia, respiration is inhibited, but alcoholic fermentation is enhanced, resulting in the inhibition of ethylene production and delayed petal senescence [14,15]. Furthermore, the expression of genes encoding the ethylene biosynthesis pathway was inhibited in hypoxic petals [16]. These results have practical significance because decreased flower ethylene biosynthesis or sensitivity prolongs the vase life of climacteric flowers, such as carnations [17].

ETHYLENE RESPONSE FACTOR (ERF), a group of plant-specific transcription factors, plays a critical role in oxygen-sensing mechanisms in plants. The ERF family, together with the APETALA2 (AP2) and RELATED TO ABI3/VP1 (RAV) families, forms the AP2/ERF superfamily because these proteins possess a conserved DNA-binding domain (AP2/ERF domain) [18]. In *Arabidopsis*, 122 members of the ERF family have been phylogenetically classified into 12 groups based on their consensus sequences [18]. Five ERF proteins in group VII have a common MCGGAI/L motif at their N-terminus, which contributes to protein stabilization in the absence of oxygen [19,20]. The N-end rule pathway enables plant cells to sense oxygen availability and alter downstream gene expression. In addition, group VII ERFs harbor members whose expression is transcriptionally induced in response to low oxygen and are called HYPOXIA-RESPONSIVE ERF (HRE) [21]. Group VII ERFs, including HREs, are essential for plant survival under oxygen-deprived conditions [20,21]. PHYTOGLOBIN (PGB) is another component of hypoxic signaling and survival [22]. In carnation flowers, hypoxia-responsive genes encoding SUCROSE SYNTHASE (SUS) and fermentative enzymes, such as PYRUVATE DECARBOXYLASE (PDC) and ALCOHOL DEHYDROGENASE (ADH), have been identified [23,24]. However, no information is available on the oxygen-sensing and signaling components, including group VII ERF, in carnations.

In this study, MAP lowered the oxygen concentration in the bag and slowed the opening of cut carnation flowers. Changes in AEC and the expression of hypoxia-related genes were investigated in petals from florets stored under MAP, with genes encoding HRE and PGB newly identified using the carnation genome database [25]. Based on the results of these investigations and the vase life of flowers after MAP storage, the effects of low-oxygen conditions accompanied by MAP are discussed.

## 2. Results

### 2.1. Effects of MAP on Opening and Vase Life of Cut Carnation Flowers

**Figure 1:**
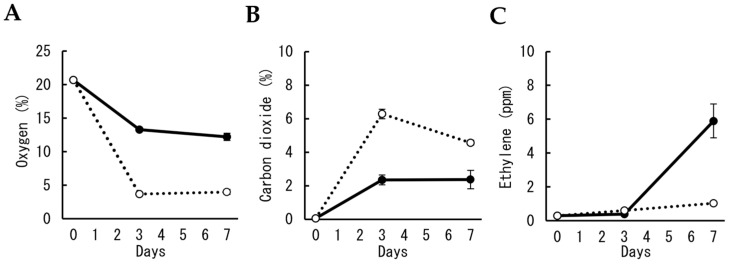
Changes in gas concentrations in polypropylene bags during MAP storage of cut carnation flowers. Concentrations of oxygen (**A**), carbon dioxide (**B**), and ethylene (**C**) in the bag were compared between the different numbers of cut flowers in a bag, two (solid line) and ten (dotted line). Data are expressed as the mean ± SE of six bags.

In the first experiment, the effect of the numbers of cut flowers on the gas concentration in polypropylene bags for MAP was investigated. The oxygen concentration in bags containing two and 10 cut flowers decreased after three days to approximately 13 and 4%, respectively (Figure 1A). Although carbon dioxide concentration increased to approximately 2.3% in three days in bags with two cut flowers, it increased to approximately 6% when 10 cut flowers were packed (Figure 1B). Ethylene concentration increased to approximately 6 ppm in seven days in bags with two cut flowers but was maintained at less than 1 ppm in bags with 10 cut flowers for seven days (Figure 1C).

**Figure 2:**
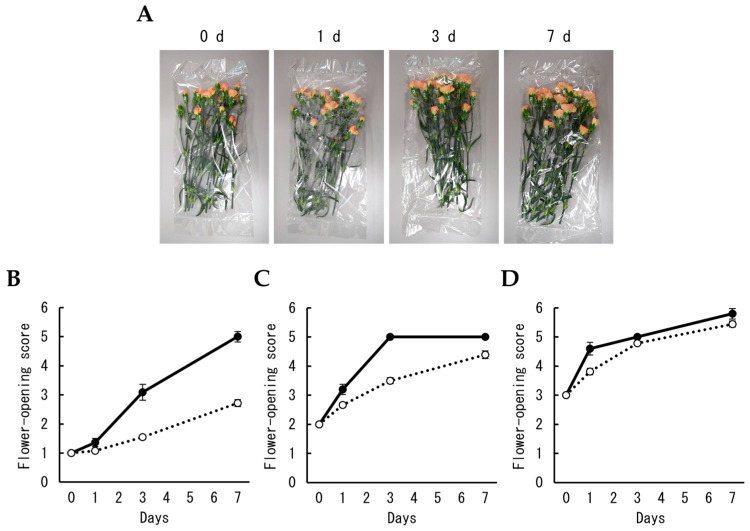
Effects of MAP on the opening of cut carnation flowers. (**A**) Appearance of cut carnation flowers during MAP storage. Changes in the opening stages of florets at stage 1 (**B**), stage 2 (**C**), and stage 3 (**D**) were monitored, respectively, and compared between two different conditions, incubation with water containing antibacterial (solid line) and MAP storage (dotted line). Data are expressed as the mean ± SE (incubation without MAP: n = 5 or 11, MAP storage: n = 29–68).

**Figure 3:**
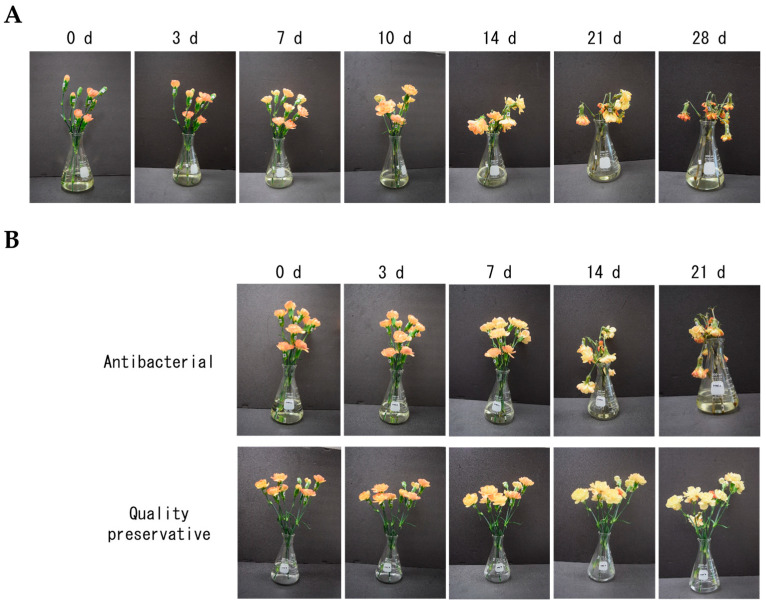
Effects of MAP on vase life of cut carnation flowers. (**A**) Cut flowers without MAP storage were incubated with water. (**B**) Cut flowers after seven days of MAP storage were incubated with water containing antibacterial (top) and with a commercial-quality preservative (bottom).

In the second experiment, the progress of flower opening during MAP storage was investigated based on the flower-opening score indexed by six opening stages [26,27]. The flower-opening score of florets at stage 1 from cut flowers incubated with water increased gradually and reached 5.0 for seven days, whereas the score during MAP storage changed more slowly and reached 2.3 for the same number of days (Figure 2A,B and Figure 3A). Although a similar trend was observed for florets at stages 2 and 3, the difference between the two conditions decreased as opening progressed (Figure 2A,C,D and Figure 3A). The petals of unpacked flowers shrank or lost tension within seven days. These results showed that flower opening was suppressed during MAP storage.

The vase life of cut flowers was also evaluated after MAP storage for seven days. When cut flowers were incubated with water-containing antibacterial agents, petal wilting and stem breakage were observed 14 days after the start of incubation, regardless of MAP storage (Figure 3A,B). In contrast, incubation with a commercial-quality preservative prevented these symptoms and prolonged the vase life of cut flowers (Figure 3B). These results suggest that the vase life of cut flowers after seven days of MAP storage is comparable to that without storage, and the water uptake capacity and keeping quality of cut flowers could be improved even after MAP storage.

### 2.2. Effects of MAP on AEC in Carnation Petals

Changes in AEC levels in carnation petals were investigated based on ATP, ADP, and AMP content. After a 3-day incubation of the isolated florets under hypoxia (3% oxygen), the ATP/ADP ratio and AEC levels tended to increase compared to those before incubation (Figure S1A,B). In contrast, the content of these adenyl nucleotides decreased, and the AEC levels significantly decreased (Figure S1C). These results indicate that the AEC levels in carnation petals are affected by oxygen availability.

**Figure 4:**
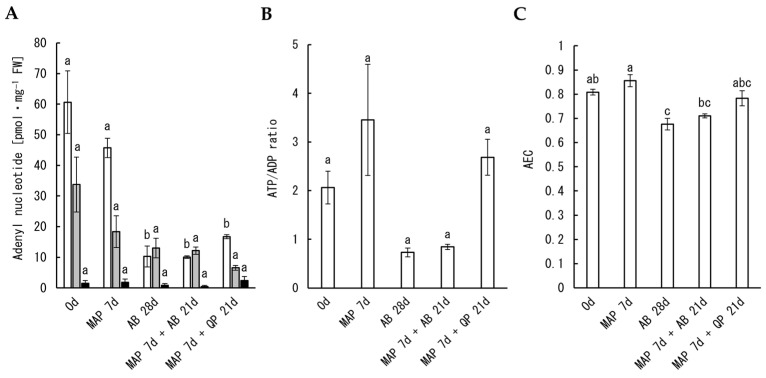
Effects of MAP and incubation with antibacterial (AB) and a commercial quality preservative (QP) on adenyl nucleotide contents, ATP/ADP ratio, and AEC in carnation petals. Contents of ATP (white bars), ADP (gray bars), and AMP (black bars) in petals were determined using a luminometric method (**A**) and used for the calculation of ATP/ADP ratio (**B**) and AEC (**C**). Data are expressed as the mean ± SE of three separate samples. Significant differences (*p* < 0.05) detected using Tukey’s multiple comparison test are indicated by different letters above the bars.

AEC levels were measured in the petals of cut carnation flowers before and after MAP storage. ATP content, ATP/ADP ratio, and AEC after MAP storage for 7 days were maintained at levels comparable to those before storage, whereas ATP content and AEC levels dropped after 28-day incubation without MAP storage (Figure 4). Similar changes were observed in flowers after a 21-day incubation following 7 days of MAP storage, with trends of improvement in the ATP/ADP ratio and AEC when a quality preservative was applied (Figure 4). Based on these results, it is suggested that the energy status of carnation petals is maintained after seven days of MAP storage.

### 2.3. Identification of the ERF Gene Family and a PGB Gene in Carnation

**Figure 5:**
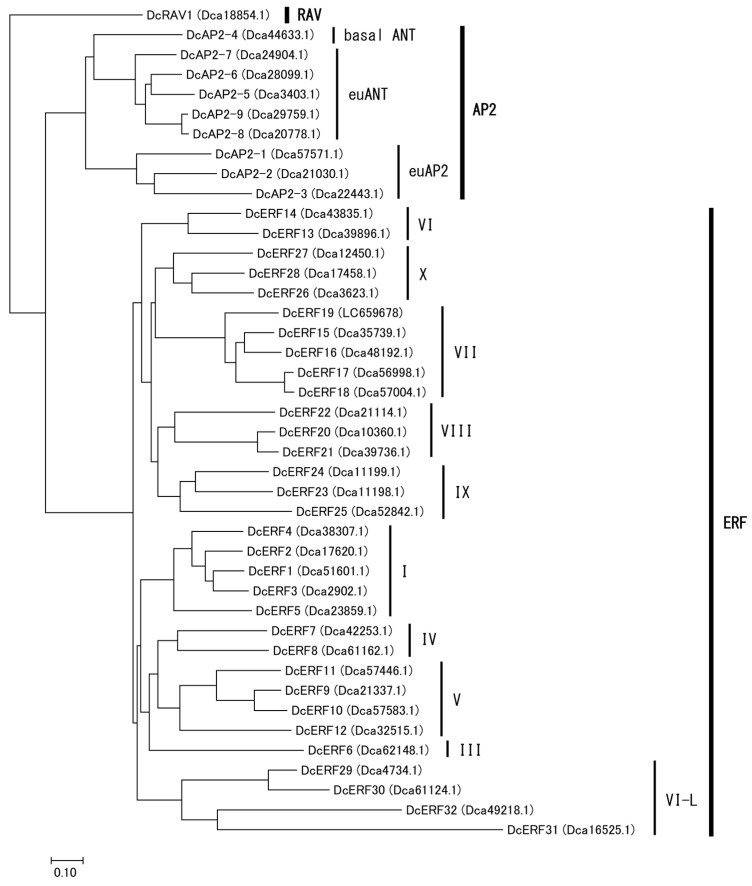
Phylogenetic tree of AP2/ERF superfamily from carnation. Amino acid sequences deduced from the AP2, RAV, and ERF genes of carnations, distinguished by ORF ID shown in parentheses, were aligned using ClustalW. The tree was constructed based on the neighbor-joining method using MEGA7.0 software.

**Figure 6:**
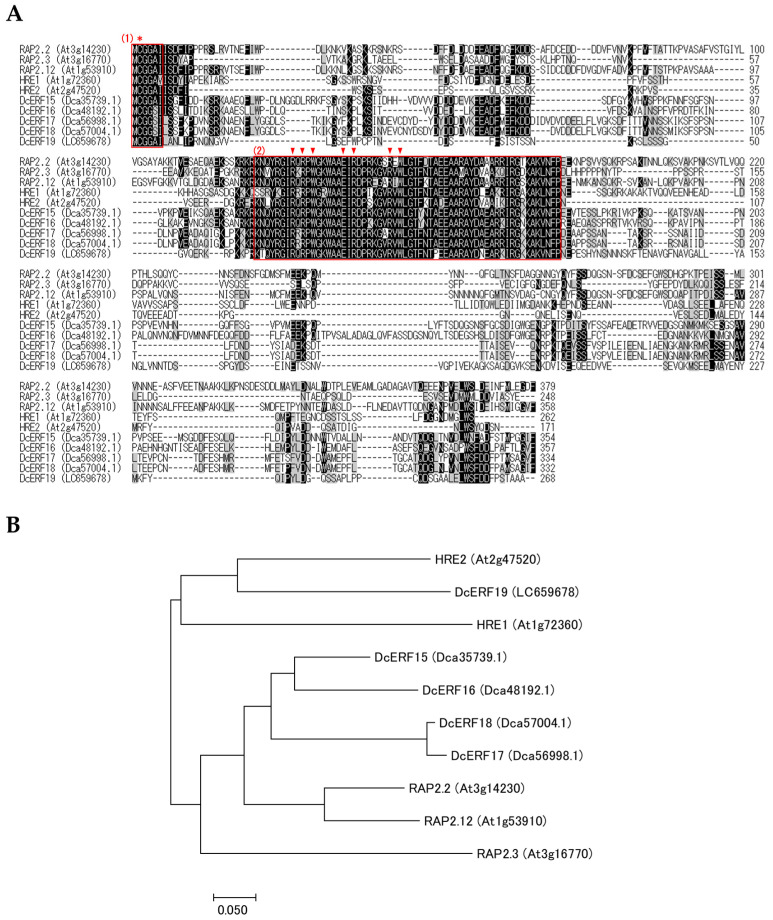
Structural and phylogenetic characterization of group VII ERF from carnation. (**A**) Multiple alignment of deduced amino acid sequences of group VII ERF from *Arabidopsis* and carnation. The sequences were aligned using ClustalW and BioEdit software. Identical or similar amino acids are indicated by a black or gray background, respectively, and gaps are indicated by dashes. The MCGGAI/L motif and AP2/ERF domain are indicated by red boxes (1) and (2), respectively. A cysteine residue oxidized in air and amino acid residues directly in contact with DNA [18] are indicated by an asterisk and arrowheads, respectively. (**B**) Phylogenetic tree of group VII ERF from *Arabidopsis* and carnation. Amino acid sequences deduced from the group VII ERF genes of *Arabidopsis* and carnation, distinguished by AGI ID or ORF ID shown in parentheses, were aligned using ClustalW. The tree was constructed based on the neighbor-joining method using MEGA7.0 software.

Homology searches were performed using the carnation genome database to identify the hypoxia-related genes. Considering that transcription factors containing the AP2/ERF domain are classified into three different lineages—AP2, RAV, and ERF—all members of the superfamily were phylogenetically confirmed simultaneously. As a result, thirty-two members of the ERF family were identified, together with nine members of the AP2 family and one RAV gene, and were named based on their subgrouping (Tables S1 and S2, Figure 5, Figures S2 and S3). Interestingly, carnation ERFs were classified into 10 of the 12 groups identified in *Arabidopsis* [18]. Groups II and Xb-L were completely absent, and only one member was found in group III. Five genes encoding group VII ERFs were identified, based on the presence of the MCGGAI/L motif. As the nucleotide sequence of Dca55626.1, deposited in the database, was incomplete, cDNA was cloned from two cultivars, and the whole coding sequence (CDS) was determined and named *DcERF19* (accession nos. LC659678 and LC659682). The phylogenetic analysis of the deduced amino acid sequences showed that DcERF19 was closely related to *Arabidopsis* HREs (Figure 6). For PGB, only one sequence (Dca3435.1) that showed homology with *PGB1* from *Arabidopsis* was found in the database (Figure S4). The cDNA sequences cloned from the two cultivars matched this reference sequence completely and were named *DcPGB1* (accession nos. LC659679 and LC659683).

### 2.4. Expression of Hypoxia-Related Genes in Petals from Florets Stored under MAP Conditions

Changes in the transcript levels of five group VII ERF genes in petals in response to ethylene treatment and hypoxia (1% oxygen) were investigated using real-time RT-PCR. Although no significant changes were observed in the transcript levels of *DcERF15* and *DcERF17*, those of *DcERF16* and *DcERF18* increased after 3 h and 12 h of ethylene treatment, respectively (Figure S5A–D). *DcERF19* transcript levels uniquely increased during hypoxic incubation (Figure S5E). These results show that *DcERF19* is the sole HRE gene in carnations.

**Figure 7:**
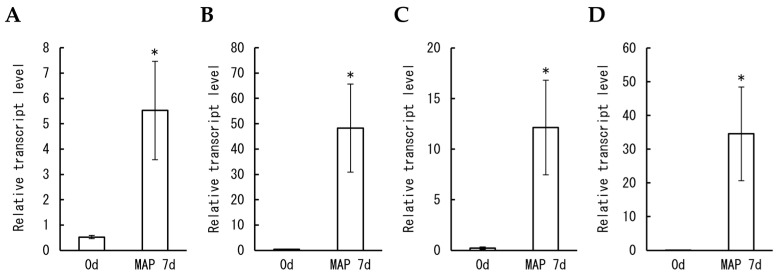
Effects of MAP on transcript levels of hypoxia-related genes in carnation petals. Relative transcript levels of *DcSUS2* (**A**), *DcADH1* (**B**), *DcERF19* (**C**), and *DcPGB1* (**D**) in petals before (stage 2) and after (stage 4) MAP storage for seven days were determined using real-time RT-PCR with *DcUbq3-7* as a standard. Data are expressed as the mean ± SE of three separate samples. Significant differences (*p* < 0.05) detected using Student’s *t*-test are indicated by an asterisk above the bars.

The transcript levels of *DcERF19* and *DcPGB1* in the petals from florets after MAP storage were investigated, as were those of *DcSUS2* (accession nos. LC659676 and LC659680) and *DcADH1* (accession nos. LC659677 and LC659681) are upregulated under hypoxia [21]. The transcript levels of all four genes were markedly increased compared to those before storage (Figure 7). *DcSUS2*, *DcADH1*, and *DcPGB1* were significantly upregulated, at least transiently, in petals from isolated florets incubated under 3% oxygen (Figure S6). These results suggested that hypoxia-responsive genes maintain enhanced expression in carnation petals under prolonged hypoxia during MAP storage.

## 3. Discussion

Dry management is a frequently adopted methodology by the cut flower supply chain, owing to reduced transport and handling costs [28], and near-ambient temperature, which is frequently used in the supply chain of developing countries [29], is employed during storage. In addition, methods for increasing the degree of certainty that a cut flower will last a minimum length of time are needed, not only for sustainability reasons but also for the expansion of the horticultural industry [11]. In this study, the effects of MAP were confirmed using cut carnation flowers. As expected, the number of packed cut flowers affected the gas concentration in the bag. Climacteric ethylene may cause petal wilting in carnation flower packaging. This can be avoided when an appropriate number of cut flowers with florets at the early opening stages are used, which might be attributed to the inhibitory effects of ethylene action through high carbon dioxide and developmental processes by low oxygen [1,16]. Decelerated flower opening during MAP storage has been demonstrated to have a favorable effect on preserving cut flower quality. This has also been confirmed in the packaging of gladiolus flowers with SA-type oxygen absorbers, which can hardly be applied to ethylene-sensitive flowers, such as carnations, because these absorbers release ethylene [6]. In this study, the vase life of cut carnation flowers was not affected by MAP storage. In addition, the loss of the ornamental value of cut carnation flowers after MAP storage was prevented by the application of a quality preservative. This indicates that cut flowers have the potential to further extend their longevity after MAP storage. Considering that both petal wilting and stem breakage were prevented, sugars and/or surfactant in the agent may have contributed to this effect, although it is difficult to determine which component was responsible. Further analysis is required to clarify the factors affecting cut flower quality following MAP.

Energy crisis is one of the concerns caused by a lack of oxygen because the balance between the production and consumption of ATP can be lost. AEC is a useful indicator of energy status [12], and this study confirmed that high AEC levels were maintained in carnation petals from florets stored under MAP conditions. This indicates that ATP production balanced its consumption when oxidative phosphorylation was inhibited [13,15]. Sugar utilization is altered in carnation petals under hypoxia [24]. In addition to the enhancement of alcoholic fermentation [14,23], sucrose degradation mediated by SUS may be involved in the altered sugar energy metabolism under prolonged hypoxia [24]. However, in carnation petals wilting in air, the AEC levels were relatively unchanged compared with the ATP content, which agrees with previous results [13]. Therefore, AEC is indicative only when petals are injured by a severe lack of oxygen but is not predictive of petal senescence. However, the florets tended to open insufficiently, and the petal color became fainter after MAP storage. The adverse effects of hypoxia on petal cell growth, depending on water uptake and pigment synthesis, could cause these phenomena, which may also depend on the growing season and cultivar of the cut carnation flowers. Since adequate flower bud opening is a key quality requirement from the customer’s point of view [30], these aspects appear to be current concerns regarding the practical utility of this technique and should be addressed in future studies.

Genes encoding HRE belonging to the group VII ERF and PGB were successfully identified and found to be upregulated in hypoxic carnation petals. Considering that these proteins are involved in oxygen sensing by interacting with other gases, such as ethylene and nitric oxide [22], DcERF19 and DcPGB1 may contribute to maintaining homeostasis in hypoxic flowers through the transcriptional regulation of genes responsible for hypoxic acclimation. Genes encoding group VII ERF have also been identified in some fruits, with their roles proposed in the deastringency process in persimmon [31] or in the bulky tissues inside which oxygen concentration could be significantly lowered during low-oxygen storage [32]. The protein stability of group VII ERF mediated by the N-end rule pathway [19,20] remains to be confirmed after the elucidation of the oxygen-sensing mechanisms during CA or MAP storage of these horticultural crops.

In the process of identifying the group VII ERF and HRE in this study, the entire picture of the AP2/ERF superfamily in the carnation genome was revealed. For AP2, a small number of members were classified into the euAP2 and AINTEGUMENTA (ANT) lineages [33]. It has been suggested that Dca21030.1 (*DcAP2-2*) belongs to the *PETALOSA* (*PET*) lineage and is a determinant of double flower traits [34,35]. The DcAP2 family is useful for understanding the molecular evolution of AP2 the family and the mechanisms underlying flower development, which is a relevant physiological process with horticultural significance in carnations. Regarding ERF, only *DcERF1* was cloned in a previous study and was reported to be preferentially expressed in petals at the early opening stages [26]. Surprisingly, the carnation ERF family consists of a minimum number of genes compared to other plant species, including *Arabidopsis*, rice, and some ornamental flowers [18,36,37]. Although members of groups II and Xb-L were completely absent, members of group VII appeared to be relatively conserved, suggesting that the latter has more fundamental roles beyond species. ERF was discovered as a transcription factor protein family that is responsive to ethylene and is a component of the ethylene signaling transcriptional cascade [38,39]. Ethylene-inducible ERF genes, such as those in petunia and rose, have been identified in other ornamental flowers [40,41]. In the present study, *DcERF16* and *DcERF18* were upregulated in response to ethylene treatment, but the ethylene responsiveness of *DcERFs* other than those in group VII is unknown. How DcERFs are involved in ethylene signaling associated with climacteric ethylene production, a characteristic trait of carnation flowers, remains to be clarified.

## 4. Materials and Methods

### 4.1. MAP and Evaluation of Vase Life of Cut Carnation Flowers

Cut flowers of spray-type carnation (*Dianthus caryophyllus* L. ‘Ekubo’) 800 mm long with five leaf pairs on a main stem were transported to our laboratory in a cardboard box without water supply at room temperature from a farm in Marugame City, Kagawa Prefecture, Japan. Following the raising of tap water, two or ten cut flowers with two to four florets at opening stages 2–4 [26] were cut into 300 mm long pieces with two leaf pairs on a main stem and packed in a bag (220 mm × 500 mm) made of polypropylene tube (0.03 mm width, 3800 cm^3^ m^−2^ d^−1^ and 9.1 g m^−2^ d^−1^ of oxygen and water vapor transmission rate, respectively, Taiyousha, Osaka, Japan), with both ends of the bag heat-sealed. The bags were stored at 23 °C in the dark for seven days. The concentrations of oxygen, carbon dioxide, and ethylene in the bags were measured using a Three Gas Analyzer F-950 (Felix Instruments, Camas, WA, USA). Following MAP storage, cut flowers were incubated with distilled water containing 200 mL of 0.2 mM 8-hydroxyquinoline sulfate (antibacterial) or the quality preservative solution (Mizuage-meijin, Hyponex Japan, Osaka, Japan) containing sugar and antibacterial and surfactant agents under continuous lighting using fluorescent lamps (photosynthetic photon flux density: 6.5 μmol m^−2^ s^−1^) at 23 °C and approximately 50% relative humidity. Vase life was evaluated based on wilting or browning symptoms of petals or stem breakage following previous studies [42,43].

### 4.2. Determination of ATP, ADP, and AMP

Florets at opening stage 4 were incubated in air, hypoxia (3% oxygen), and anoxia, as previously described [24,44]. After incubation, petals were frozen and ground using a mortar and pestle. Following a previous study [45], the resulting powder (100 mg) was suspended in 1 mL of 1.66 M perchloric acid. Followed by the centrifugation of the suspension at 1000× *g* for 15 min, 0.2 mL of 1 M (*N,N*-bis)2-hydroxyethylglycine (Bicine) and 0.35 mL of 4 M KOH were added to 0.8 mL of supernatant, of which the pH was checked and adjusted to 7.6–8.0. ATP, ADP, and AMP contents were determined via luminometric methods using a GloMax 20/20 luminometer (Promega, Madison, WI, USA) following previous studies [45,46].

### 4.3. Database Search and Sequence Analysis of Hypoxia-Related Genes

Following to a previous study [24], a homology search was performed on the Carnation DB (http://carnation.kazusa.or.jp/, accessed on 1 July 2020) using the BLASTX program. Five group VII ERF genes from *Arabidopsis thaliana*, that is, *RAP2.2* (At3g14230), *RAP2.3* (At3g16770), *RAP2.12* (At1g53910), *HRE1* (At1g72360), and *HRE2* (At2g47520), and one PGB gene from *A. thaliana*, i.e., *PGB1* (At2g16060), were selected as query sequences. A BLASTX search was performed using the identified sequences as query sequences against nucleotides, expressed sequence tags (EST), and transcriptome shotgun assembly (TSA) from carnations [47,48] on the NCBI website (https://blast.ncbi.nlm.nih.gov/Blast.cgi, accessed on 1 October 2020) to obtain evidence of gene expression. Multiple alignments were created with ClustalW using BioEdit software. Phylogenetic trees were constructed using the neighbor-joining method in MEGA7.0 software.

### 4.4. RNA Extraction, cDNA Cloning, and Real-Time RT-PCR Analysis

Total RNA was extracted from frozen petal samples as previously described [24]. cDNA was then synthesized from 1 μg of RNA using ReverTra Ace reverse transcriptase (Toyobo, Osaka, Japan) and Oligo dT-Adaptor Primer (5′-GTT TTC CCA GTC ACG ACT TTT TTT TTT TTT TTT TTT T-3′) and used as a template for RT-PCR. An unidentified portion of Dca55626.1 (*DcERF19*) was obtained via the 3′ rapid amplification of cDNA ends using KOD plus DNA polymerase (Toyobo) and M13PrimerM4 (Table S3). The coding sequences (CDS) of *DcSUS2*, *DcADH1*, *DcERF19*, and *DcPGB1* were amplified using gene-specific primer pairs. The amplified cDNA fragments were subcloned into a pGEM-T Easy Vector (Promega, Madison, WI, USA) and sequenced using a 3130 Genetic Analyzer (Applied Biosystems, Foster City, CA, USA). The nucleotide sequences obtained were deposited in the DDBJ/EMBL/GenBank databases under accession nos. LC659676–LC659683.

For real-time RT-PCR, cDNA fragments of the target genes were amplified from each template cDNA sample with gene-specific primer pairs (Table S3) using the Fast SYBR Green reagent in the StepOne Plus Real-Time PCR System (Applied Biosystems). The PCR conditions were 95 °C for 20 s, 45 cycles of 95 °C for 3 s, and 60 °C for 30 s. Plasmids containing cloned target sequences were used as templates for quantification standards. *DcUbq3-7* [49] was used as an internal standard.

## 5. Conclusions

A MAP condition that maintains flower quality to some extent has been described for cut carnation flowers. The energy status of carnation petals and the potential for prolonging flower longevity were maintained after seven days of MAP storage. The enhanced expression of genes involved in hypoxic signaling and metabolism was observed after MAP storage, suggesting their role in maintaining the homeostasis and quality of floral tissues. More practical aspects of the MAP and global molecular responses to low-oxygen conditions in ornamental flowers should be the focus of future research. Transcriptomic analysis is currently in progress to uncover the crosstalk between low oxygen and ethylene, where the carnation serves as a promising flower material with the accumulated knowledge of postharvest physiology and genomic information.

## Data Availability

All data generated in this study are included in the article and its supplemental data files. The nucleotide sequences obtained are available in the DDBJ/EMBL/GenBank databases with accession nos. LC659676–LC659683.

## Supplementary Materials

The following supporting information can be downloaded at: https://www.mdpi.com/article/10.3390/plants12142738/s1, Table S1: AP2 and RAV genes identified in Carnation DB; Table S2: ERF genes identified in Carnation DB; Table S3: Primers used for cDNA cloning and real-time RT-PCR; Figure S1: Effects of hypoxia and anoxia on adenyl nucleotide contents, ATP/ADP ratio, and AEC in carnation petals; Figure S2: Multiple alignment of deduced amino acid sequences of AP2 and RAV from carnation; Figure S3: Multiple alignment of deduced amino acid sequences of ERF from carnation; Figure S4: Multiple alignment of deduced amino acid sequences of PGB from *Arabidopsis* and carnation; Figure S5: Effects of ethylene and hypoxia on transcript levels of group VII ERF genes in carnation petals; and Figure S6: Effects of hypoxia on transcript levels of hypoxia-related genes in carnation petals.
