# Supplementary material for: Low-Oxygen Responses of Cut Carnation Flowers Associated with Modified Atmosphere Packaging
Source: Plants (Basel). 2023 Jul 23;12(14):2738. doi: 10.3390/plants12142738 (PMC10386211; doi:10.3390/plants12142738)
Supplement: Supplementary file 1 [file plants-12-02738-s001.zip › plants-2487204-supplementary.pdf]

Table S1. AP2 and RAV genes identified in Carnation DB.

Gene Name	ORF ID	Scaffold ID	CDS (bp)	Amino Acid Residue Number	Information on Expression
DcAP2-1	Dca57571.1	scaffold83	1782	594	EST [47]
DcAP2-2	Dca21030.1	scaffold2131	1071	357	EST [47]
DcAP2-3	Dca22443.1	scaffold225	1662	554	EST [47]
DcAP2-4	Dca44633.1	scaffold515	1365	455	EST [47]
DcAP2-5	Dca3403.1	scaffold1120	1884	628	EST [47]
DcAP2-6	Dca28099.1	scaffold28	1491	497	EST [47]
DcAP2-7	Dca24904.1	scaffold248	1716	572	EST [47]
DcAP2-8	Dca20778.1	scaffold211	1431	477	EST [47]
DcAP2-9	Dca29759.1	scaffold297	1887	629	EST [47]
DcRAV1	Dca18854.1	scaffold1983	1086	362	EST [47]

Table S2. ERF genes identified in Carnation DB.

Gene Name	ORF ID	Scaffold ID	CDS (bp)	Amino Acid Residue Number	Information on Expression
DcERF1	Dca51601.1	scaffold67	813	271	Nucleotide [26], EST [47], TSA [48]
DcERF2	Dca17620.1	scaffold19	963	321	EST [47]
DcERF3	Dca2902.1	scaffold110	894	298	EST [47], TSA [48]
DcERF4	Dca38307.1	scaffold41	864	288	-
DcERF5	Dca23859.1	scaffold2381	1218	406	-
DcERF6	Dca62148.1	scaffold9805	552	184	EST [47]
DcERF7	Dca42253.1	scaffold4737	816	272	-
DcERF8	Dca61162.1	scaffold9463	915	305	-
DcERF9	Dca21337.1	scaffold216	723	241	-
DcERF10	Dca57583.1	scaffold830	738	246	EST [47], TSA [48]
DcERF11	Dca57446.1	scaffold8257	576	192	-
DcERF12	Dca32515.1	scaffold33	585	195	-
DcERF13	Dca39896.1	scaffold434	939	313	EST [47]
DcERF14	Dca43835.1	scaffold50	951	317	TSA [48]
DcERF15	Dca35739.1	scaffold373	1062	354	EST [47], TSA [48]
DcERF16	Dca48192.1	scaffold592	1071	357	EST [47], TSA [48]
DcERF17	Dca56998.1	scaffold813	1002	334	TSA [48]
DcERF18	Dca57004.1	scaffold813	996	332	EST [47], TSA [48]
DcERF19	Dca55626.1	scaffold777	804*	268*	-
DCERF20	Dca10360.1	scaffold14520	645	215	EST [47]
DcERF21	Dca39736.1	scaffold4310	612	204	EST [47], TSA [48]
DcERF22	Dca21114.1	scaffold214	789	263	EST [47], TSA [48]
DcERF23	Dca11198.1	scaffold15	945	315	EST [47], TSA [48]
DcERF24	Dca11199.1	scaffold15	801	267	-
DcERF25	Dca52842.1	scaffold7	1557	519	TSA [48]
DcERF26	Dca3623.1	scaffold113	1221	407	EST [47]
DcERF27	Dca12450.1	scaffold157	978	326	EST [47], TSA [48]
DcERF28	Dca17458.1	scaffold189	1005	335	TSA [48]
DcERF29	Dca4734.1	scaffold1179	1017	339	EST [47], TSA [48]
DcERF30	Dca61124.1	scaffold945	915	305	-
DcERF31	Dca16525.1	scaffold1819	1938	646	TSA [48]
DcERF32	Dca49218.1	scaffold6121	1479	493	-

* Data were obtained from the cDNA sequences cloned from ‘West Diamond’ (LC659678) and ‘Ekubo’ (LC659682).

Table S3. Primers used for cDNA cloning and real-time RT-PCR.

**Gene**	**Forward Primer (5' to 3')**	**Reverse Primer (5' to 3')**
cDNA cloning (3' RACE)		
DcERF19	CCCGAAAGCCATTACAACAGC	GTTTTCCCAGTCACGAC*
cDNA cloning (whole CDS)		
DcSUS2	CAGAAAATGGCAAGTCGTTTGAC	CGACCAGCGGAGAACACGTA
DcADH1	TAATGTCGAGTACCGCCGGACAAG	GATGTCGATTTCAAGCGTCC
DcERF19	CATGTGTGGTGGTGCAATTTTAGCCA	GCAGCTTATCTTTGTTGCAAACA
DcPGB1	GAGGGAAGCAAACTAACATGG	CCGGAGATGAACAAGCAGAA
Real-time RT-PCR		
DcERF15	GGATGGTCTGACCAATGTGG	CGAACAGATGACCCTGCGAA
DcERF16	GCTAGCGAGTTTTCTCAGGA	GCTCGGAAATCCATTGCCAA
DcERF17	GAGACGTCGTTCGTGGACGA	GCGATAACAGATACATGTCTGTAGAC
DcERF18	GAGACGCCATTCGTGGACAA	ACGATAACAGATACACGTCCATC
DcERF19	GCTGCGTAAAAACTGTTAGG	GCAGCTTATCTTTGTTGCAAACA
DcSUS2	GGCTGCTAACTCTTGCGGGT	CGACCAGCGGAGAACACGTA
DcADH1	CAAGCCTAGAACCGACATAC	GATGTCGATTTCAAGCGTCC
DcPGB1	CTGCGATTCAACTGCGAGAG	GCAGTGCTTCTTTCACAACC
DcUbq3-7	GTTGTTGGTTTCAGGGCTGGTTTG	CTACGGTAATTGAGAATTCACACCGAAATG

* M13PrimerM4

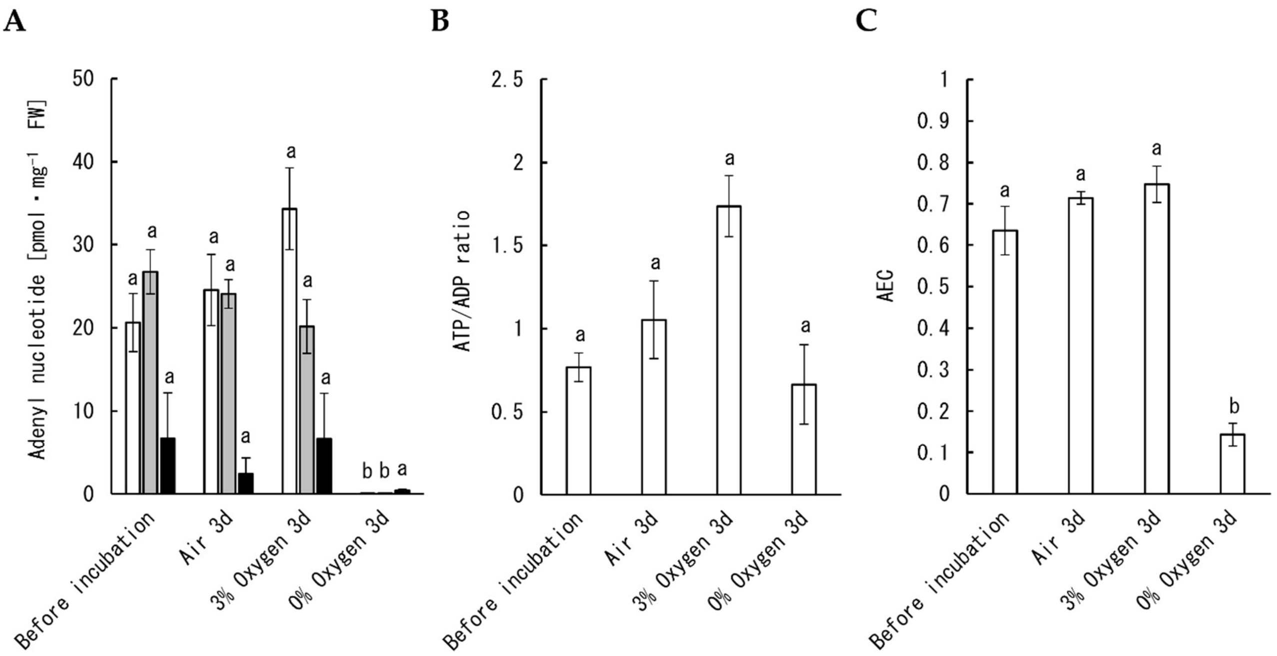

Figure S1. Effects of hypoxia and anoxia on adenyl nucleotide contents, ATP/ADP ratio, and AEC in carnation petals. Contents of ATP (white bars), ADP (gray bars), and AMP (black bars) in petals before and after incubation in air, and under hypoxia (3% oxygen) and anoxia (0% oxygen) for three days were determined using a luminometric method (**A**), and used for calculation of ATP/ADP ratio (**B**) and AEC (**C**). Data are expressed as the mean ± SE of three separate samples. Significant differences ($p < 0.05$) detected using Tukey's multiple comparison test are indicated by different letters above the bars.

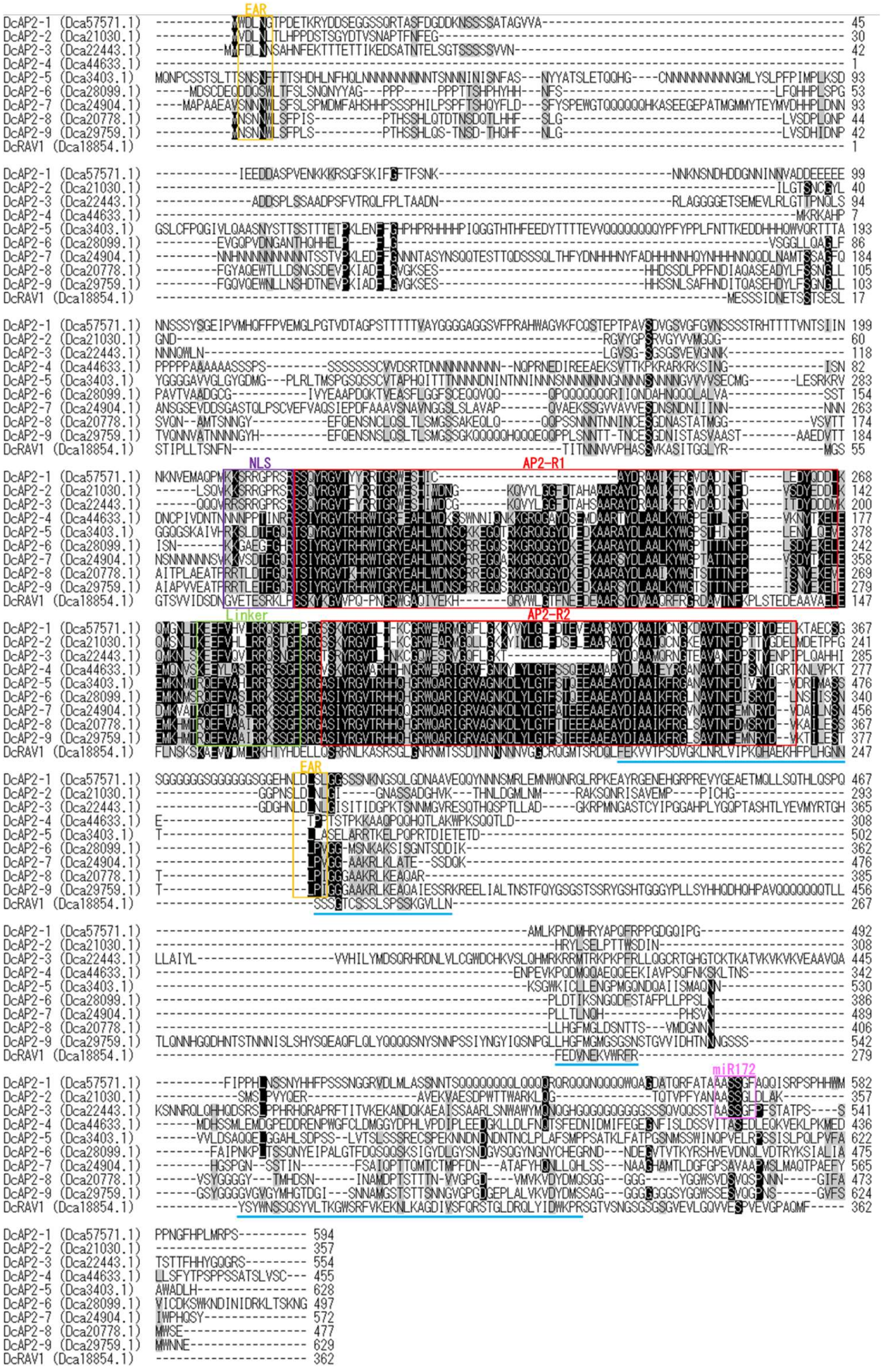

Figure S2. Multiple alignment of deduced amino acid sequences of AP2 and RAV from carnation. The sequences were aligned using ClustalW and BioEdit software. Identical or similar amino acids are indicated by a black or gray background, respectively, and gaps are indicated by dashes. The two AP2 (or ANT) domains, the EAR motif-like sequences, a putative nuclear localization signal (NLS) motif, a liker domain and the sequence derived from the target site for miR172 binding described in previous studies [33,34] are indicated by color-coded boxes. The B3-like domain conserved in RAV is indicated by blue underlines.

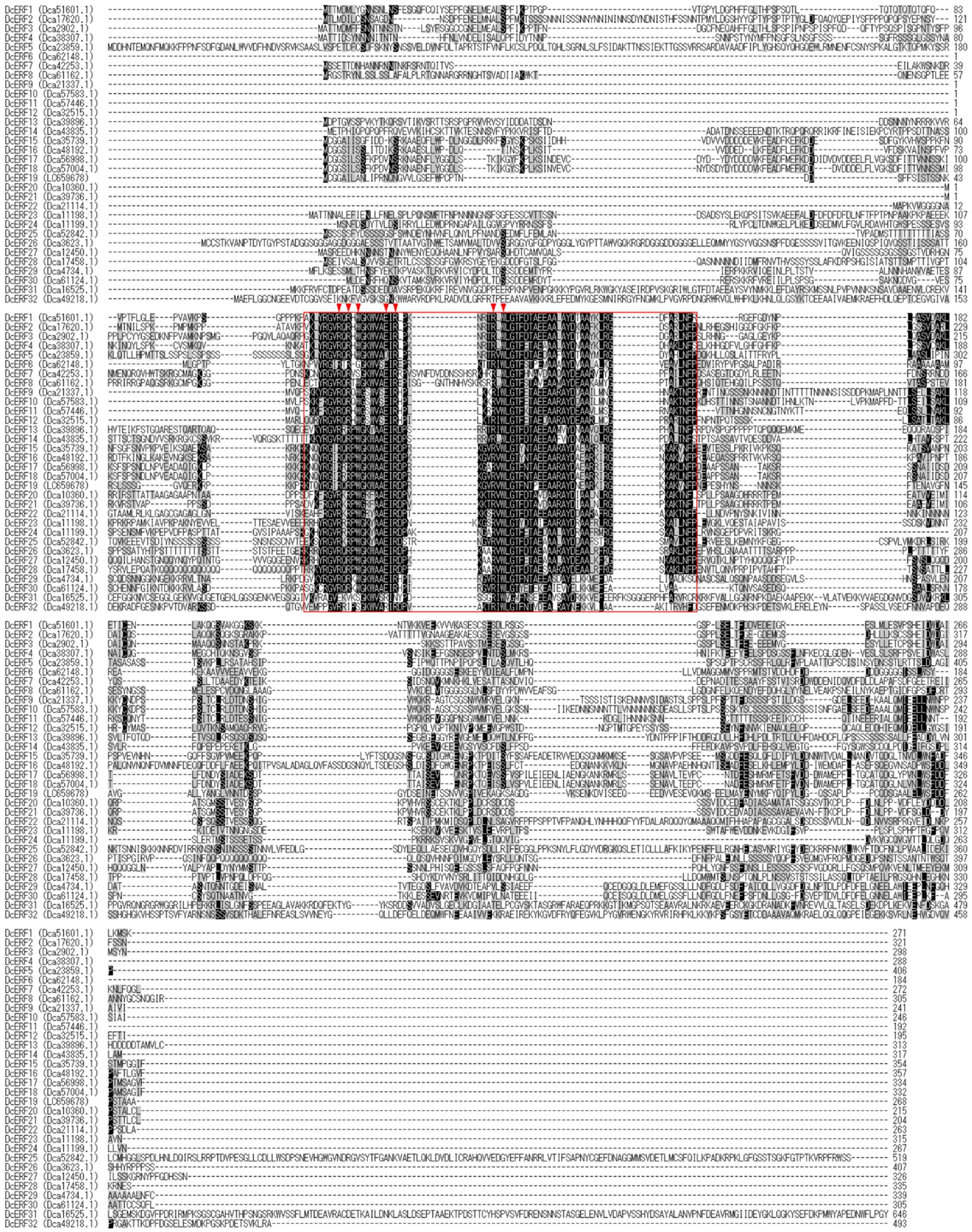

Figure S3. Multiple alignment of deduced amino acid sequences of ERF from carnation. The sequences were aligned using ClustalW and BioEdit software. Identical or similar amino acids are indicated by a black or gray background, respectively, and gaps are indicated by dashes. AP2/ERF domain and amino acid residues directly contact with DNA [18] are indicated by a box and arrowheads, respectively.

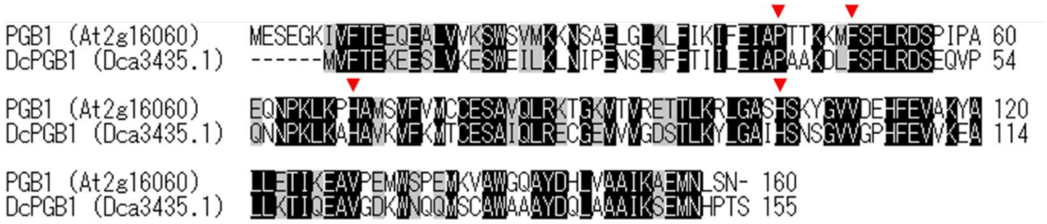

Figure S4. Multiple alignment of deduced amino acid sequences of PGB from *Arabidopsis* and carnation. The sequences were aligned using ClustalW and BioEdit software. Identical or similar amino acids are indicated by a black or gray background, respectively, and gaps are indicated by dashes. The conserved amino acid residues involved in heme and ligand binding are indicated by arrowheads.

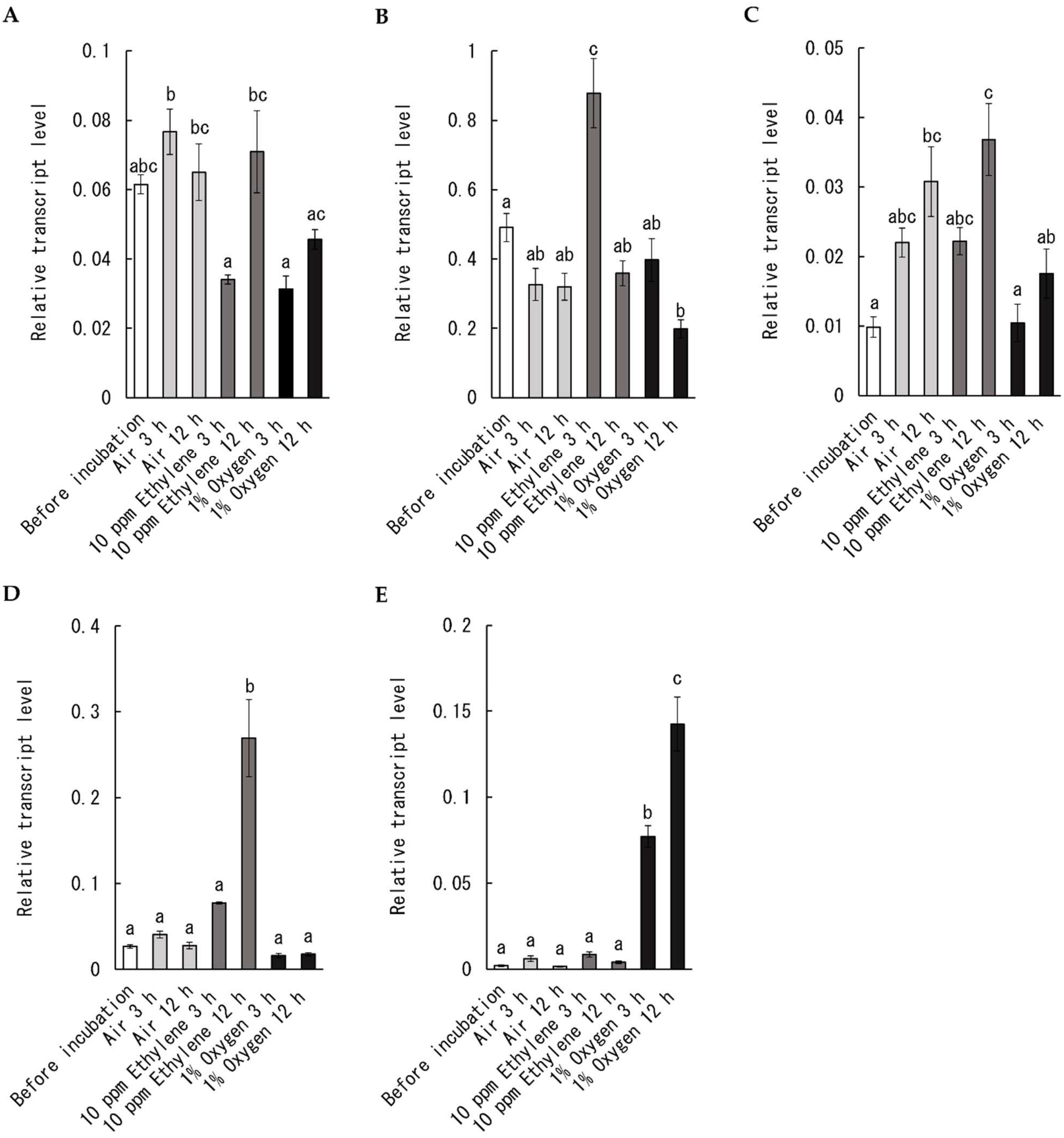

Figure S5. Effects of ethylene and hypoxia on transcript levels of group VII ERF genes in carnation ('West Diamond') petals. Relative transcript levels of *DcERF15* (**A**), *DcERF16* (**B**), *DcERF17* (**C**), *DcERF18* (**D**), and *DcERF19* (**E**) in petals before and after incubation in air, and under 10 ppm ethylene and hypoxia (1% oxygen) for 3 and 12 h were determined using real-time RT-PCR with *DcUbq3-7* as a standard. Data are expressed as the mean ± SE of three separate samples. Significant differences ($p < 0.05$) detected using Tukey's multiple comparison test are indicated by different letters above the bars.

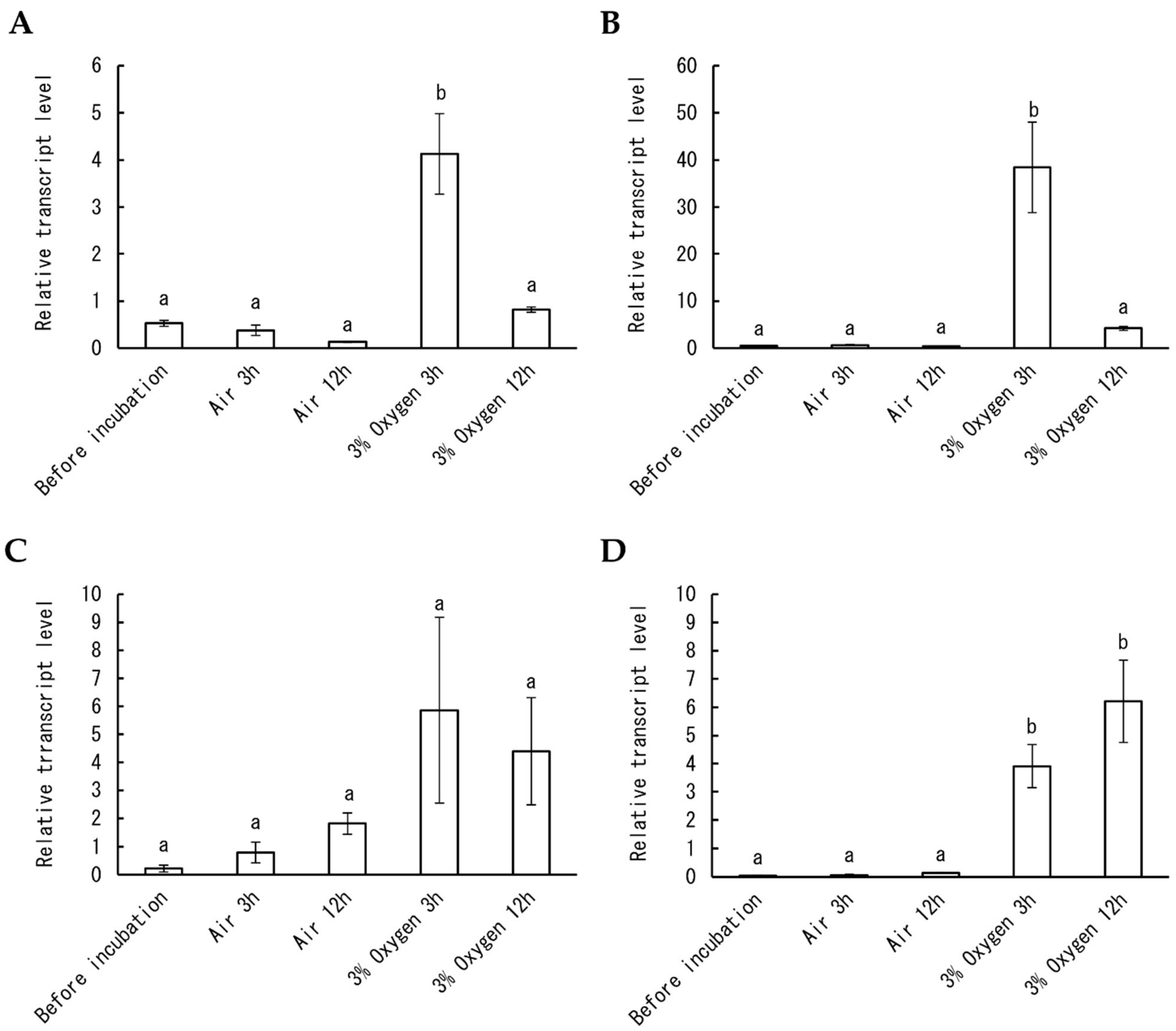

Figure S6. Effects of hypoxia on transcript levels of hypoxia-related genes in carnation petals. Relative transcript levels of *DcSUS2* (**A**), *DcADH1* (**B**), *DcERF19* (**C**), and *DcPGB1* (**D**) in petals before and after incubation in air, and under hypoxia (3% oxygen) for 3 and 12 h were determined using real-time RT-PCR with *DcUbq3-7* as a standard. Data are expressed as the mean ± SE of three separate samples. Significant differences ($p < 0.05$) detected using Tukey's multiple comparison test are indicated by different letters above the bars.
